# Oxygen, secreted proteins and small RNAs: mobile elements that govern anther development

**DOI:** 10.1007/s00497-020-00401-0

**Published:** 2021-01-25

**Authors:** Stefanie Dukowic-Schulze, Karina van der Linde

**Affiliations:** Department of Cell Biology and Plant Biochemistry, University of Regensburg, Regensburg, Germany

**Keywords:** Cell–cell communication, Gradient, Hypoxia, Ligand, Receptor, PhasiRNA

## Abstract

Correct anther development is essential for male fertility and subsequently agricultural yield. Defects in anther development range from the early stage of stamen formation until the late stage of tapetum degeneration. In particular, the specification of the four distinct somatic layers and the inner sporogenous cells need perfect orchestration relying on precise cell–cell communication. Up to now, several signals, which coordinate the anther´s developmental program, have been identified. Among the known signals are phytohormones, environmental conditions sensed via glutaredoxins, several receptor-like kinases triggered by ligands like MAC1, and small RNAs such as miRNAs and the monocot-prevalent reproductive phasiRNAs. Rather than giving a full review on anther development, here we discuss anther development with an emphasis on mobile elements like ROS/oxygen, secreted proteins and small RNAs (only briefly touching on phytohormones), how they might act and interact, and what the future of this research area might reveal.

## Introduction

Anthers are intricately formed reproductive structures which enable the creation and dispersal of male gametophytes. Anthers are thus an important factor for fertility and agronomic yield. Since anthers and anther defects are easily visible particularly in maize, early observations identified multiple classical genes required for anther development and male meiosis (partly re-analyzed and summarized by Timofejeva et al. 2013).

In general, studies provided descriptive insights about the signals orchestrating timely formation of certain anther layers, their redifferentiation, and about synchronized development of the different layers and the archesporial cells which develop into meiocytes and finally pollen in the center of the anthers. Key players and developmental programs for early anther development have been extensively studied and described (reviewed in: Timofejeva et al. 2013; Kelliher et al. 2014; Walbot and Egger 2016; van der Linde and Walbot 2019) (Table 1). Other studies gave insight into late anther and pollen development (for example, see: Dickinson and Bell 1976; Schreiber et al. 2004), and by now also provide a sound understanding about involved transcription factors and components for the tapetal cell death and pollen wall formation (for example, Huo et al. 2020; Xiong et al. 2020; Uzair et al. 2020; Lu et al. 2020). A recurring observation points to the high importance of the tapetum layer for successful pollen maturation and release (Cigan et al. 2001; Ito and Shinozaki 2002; Albrecht et al. 2005; Colcombet et al. 2005).

**Table 1:** Overview of putative mobile elements in anther development

Mobile element category	Species	Mobile element	Downstream component(s)	Mutant phenotype	Core findings/conclusions	Reference(s)
Oxygen/ hypoxia	*Z. mays*	(Oxygen)	MSCA1 (Glutaredoxin)	*msca1:* no AR cells	AR cell initiation needs hypoxia, MSCA1 part of pathway	Kelliher and Walbot (2012)
	*O. sativa*	Oxygen)	MIL1 (Glutaredoxin)	*mil1:* defects only after AR, EN + SPC formation		Hong et al. (2012b)
	*A. thaliana*	(Oxygen)	ROXY1/2 (Glutaredoxin)	*roxy1/2*: no P/SPCs (?), no pollen release, but meiosis initiates	Regulation of bZIP/TGA transcription factors by ROXY1/2	Xing and Zachgo (2008), Xing et al. (2005), Li et al. (2009), Li et al. (2011)
Secreted peptide	*Z. mays*	MAC1	MSP1 (RLK)	*mac1*: excess of AR cells	MAC1 secreted by AR cells, recognized by MSP1 in surrounding L2 cells, then EN + SPC formation	Wang et al. (2012a), van der Linde et al. (2018a)
	*O. sativa*	TDL1A	MSP1 (RLK)	*tdl1a* + *msp1*: ovule and anther defects; excess of AR cells, no TP, no ML, sterile	TDL1A-MSP1 module limits number of AR cells; induces TGA transcription factor and glutaredoxin	Nonomura et al. (2003), Yang et al. (2016)
	*A. thaliana*	TPD1	EMS1/EXS (RLK)	*tpd1 and ems1/exs:* excess AR cells, no TP, sterile	TPD1 secreted by AR cells, bound by EMS1/EXS in preTP cells, for SPC division for TP formation	Yang et al. (2003), Huang et al. (2016)
Secreted peptide	*A. thaliana*	?	ERL1/ERL2 (RLK)	*er/erl1/erl2* triple: partly absent/defective anthers, no dehiscence	ER/ERL1/ERL2 act in a pathway for anther cell division and differentiation	Hord et al. (2008)
Secreted peptide	*A. thaliana*	?	BAM1/BAM2 (RLK)	*bam1/bam2* double: only AR-like cells (fewer and larger), no internal somatic cells	BAM1 + 2 might have an early and late function, in PPC formation and PMC development support	Hord et al. (2006)
Secreted peptide	*A. thaliana*	?	SERK1/SERK2 (RLK)	*serk1/serk2* double: absent TP, microspore abortion, sterile	SERK1/2 act in pathway for TP formation	Colcombet et al. (2005)
Secreted peptide	*A. thaliana*	?	RPK2 (RLK)	*rpk2*: no ML	ML is needed for proper TP development and PCD; RPK2 part of pathway	Mizuno et al. (2007)
Secreted peptide	*O. sativa*	?	TMS10, TMS10L (RLK)	*tms10/tms10l:* no tapetum PCD, sterile at high temperature	TMS10/TMS10L act through their kinase activity in a signaling pathway for PCD	Yu et al. (2017)
Secreted peptide	*A. thaliana*	CLE1, 7, 11, 12, 13, 25	?		CLE's with particular expression patterns, suitable for processes and RLKs above	Jun et al. (2010)
Small RNAs & hormone	*A. thaliana*	Auxin; miR167; further tasiARFs?	ARF6 + 8	*arf6 arf8* double, *miR167* overexpression: short stamen, larger connective cells, smaller vascular bundles, no dehiscence	miR167-ARF6/8 module needed for proper anther dehiscence	Nagpal et al. (2005); Wu et al. (2006)
Small RNAs	*A. thaliana*	miR165/6	PHB (transcription factor) → SPL/NZZ	miR154/5 overexpression: two instead of four lobes; spl/nzz: no EN + TP formation, no initiation of meiosis	miR165/6 has an early role in anther structure formation, and a later one in cell differentiation	Li et al. (2019a), Yang et al. (1999), Liu et al. (2009b)
Small RNAs	*A. thaliana*	miR156	SPLs (transcription factors)	miR156 overexpression in spl8 mutant (targeting other SPLs): sterile, almost no differentiation	miR156 supports timely decay of SPL transcription factors for regulation of cell division, differentiation and specification in early anther development	Xing et al. (2010)
Small RNAs	*A. thaliana*	het-siRNAs???	AGO2 → HXK	*ago*2 KO/ *HXK* overexpression: anther defects, ROS accumulation, premature PCD of the TP	negative regulation of ROS production through HXK by AGO2 and unknown sRNAs with unknown origins	Zheng et al. (2019a, b)
Small RNAs	*O. sativa*	phasiRNAs	MEL1 (AGO5)	*mel1:* defects in PMC and tapetum development	MEL1 binds 21nt phasiRNAs with mostly 5′C and acts in a pathway for large-scale meiotic chromosome reprogramming	Nonomura et al. (2007), Komiya et al. (2014), Liu and Nonomura (2016)
Small RNAs	*Zea mays*	phasiRNAs	AGO18b	*ago18b*: more spikelets, defect in meiosis II (still fertile)	AGO18b binds 21nt phasiRNAs with mostly 5′U, and weaker to 24nt phasiRNAs with mostly 5′A; AGO18b is expressed at inflorescence meristems, tapetum and PMCs and might interact with a miR166-HD-ZIP transcription factor module	Zhai et al. (2014), Sun et al. (2018), Sun et al. (2019)

Mobile element category	Species	Mobile element	Upstream component(s)	Mutant phenotype	Core findings/ conclusions	Reference(s)
Small RNAs	*A. thaliana*	? miRNAs/ tasiRNAs	DCL1 (biosynthesis)	*dcl1: partly abnormal anthers, less lobes; viable pollen*	sRNAs play a role in early anther formation	Jacobsen et al. (1999)
	*O. sativa*	miR2118 + miR2275	DCL1 (biosynthesis)	*dcl1: strong decrease of miR2118 and miR2275 for phasiRNA production*	DCL1 is needed for biogenesis of the phasiRNA triggers miR2118 and miR2275	Song et al. (2012)
	*Z. mays*	? miRNAs/ tasiRNAs/ phasiRNAs	DCL1 (biosynthesis)	*dcl1: sterile, defects in maturation and dehiscence*	sRNAs play a role in later anther development	Field and Thompson (2016)
Small RNAs	*O. sativa*	21nt phasiRNAs	miR2118 (trigger)	*miR2118: male and female sterile, anther wall defects (EPI, ML, TP)*	21nt phasiRNAs in anther walls have mostly 5′U, and seem important for epidermis differentiation, and maturation of ML and TP	Araki et al. (2020)
Small RNAs	*Z. mays*	21nt phasiRNAs	OCL4 (tx factor)	*ocl4: sterile, duplicated EN*	Gene expression regulated by OCL4 in the epidermis is needed for production of 21nt phasiRNAs	Wang et al. (2012a), Zhai et al. (2015)
Small RNAs	*O. sativa*	21nt phasiRNAs (/tasiRNAs)	DCL4 (biosynthesis)	*dcl4: strong reduction of 21nt phasiRNAs in panicles*	DCL4 is needed for biogenesis of 21nt phasiRNAs	Song et al. (2012)
Small RNAs	*O. sativa*	24nt phasiRNAs (/tasiRNAs)	DCL3b* (biosynthesis)	*dcl3b: strong reduction of 24nt phasiRNAs in panicles*	DCL3b is needed for biogenesis of 24nt phasiRNAs	Song et al. (2012)
Small RNAs	*Z. mays*	24nt phasiRNAs	DCL5* (biosynthesis)	*dcl5: short anthers, defective TP, temperature-sensitive male sterility*	DCL5 is needed for biogenesis of 24nt phasiRNAs; 24nt phasiRNAs are critical for male fertility at regular temperature, likely via supporting TP differentiation	Teng et al. (2020)

*Rice DCL3b and maize DCL5 are homologs; maize DCL3b was renamed into DCL5

**Fig. 1:**
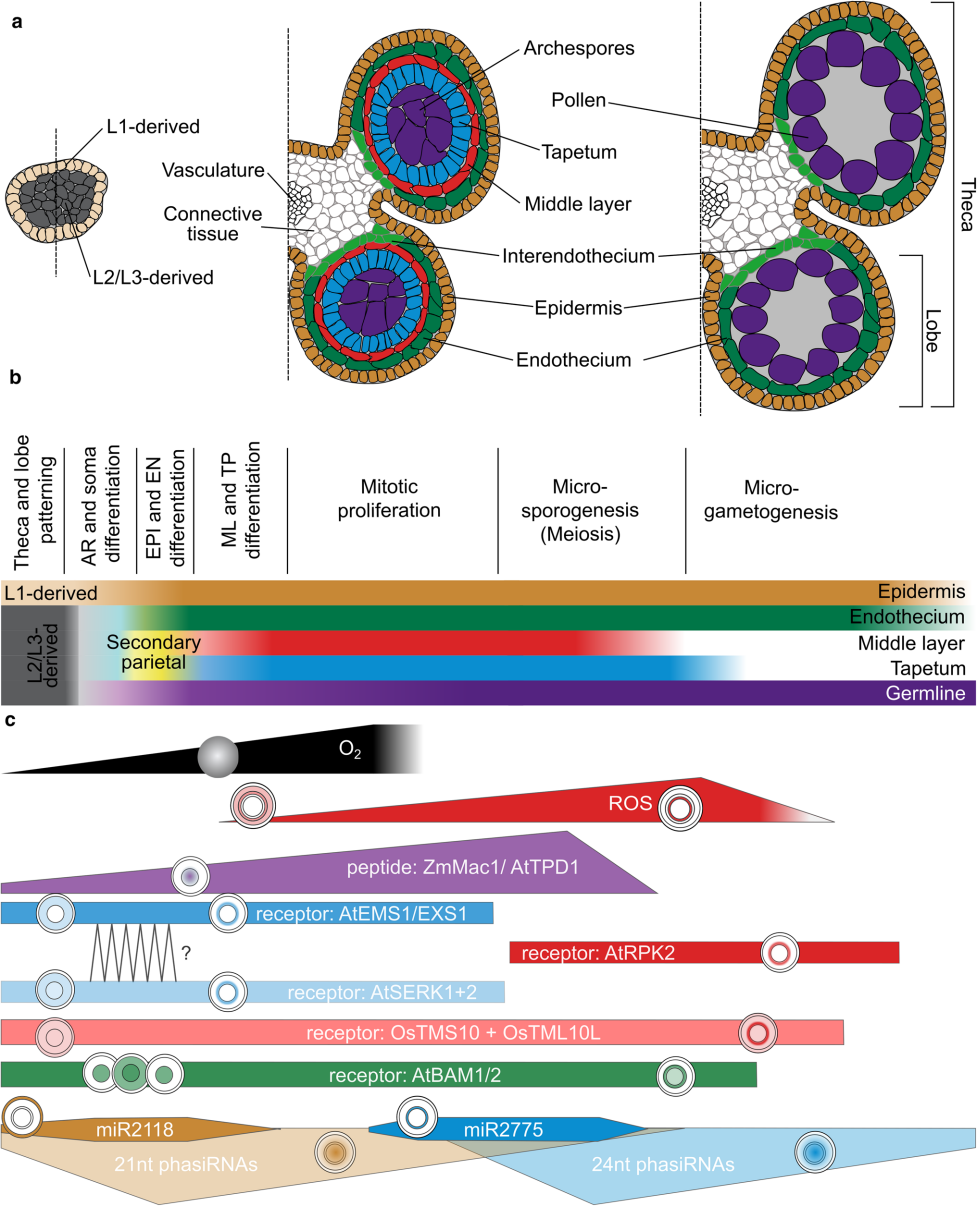
Schematic developmental progression of maize anthers and key mobile elements involved in the process. **a** Schematic cross sections or half-cross section of different anther developmental stages, ranging from before lobe specification, through mitotic proliferation to microgametogenesis. All anther cells date back to L1-d, L2-d cells and in dicots L3-d cells. L1-d become epidermis, while L2-d/L3-d cells form the vasculature, connective tissue and all lobe tissues. **b** Schematic timeline of anther lobe development. **c** Mobile elements known to occur during anther development. The approximate timing and levels of occurrence are depicted schematically by filled areas. Circles represent anther lobes, with gradients or layer-specific location shown by corresponding colors. Colors used are selected due to the colors in panel b, with mobile elements arising or occurring in the respective color-coded layers. AR: archesporial, EN: endothecium, EPI: epidermis, TP: tapetum

**Fig. 2:**
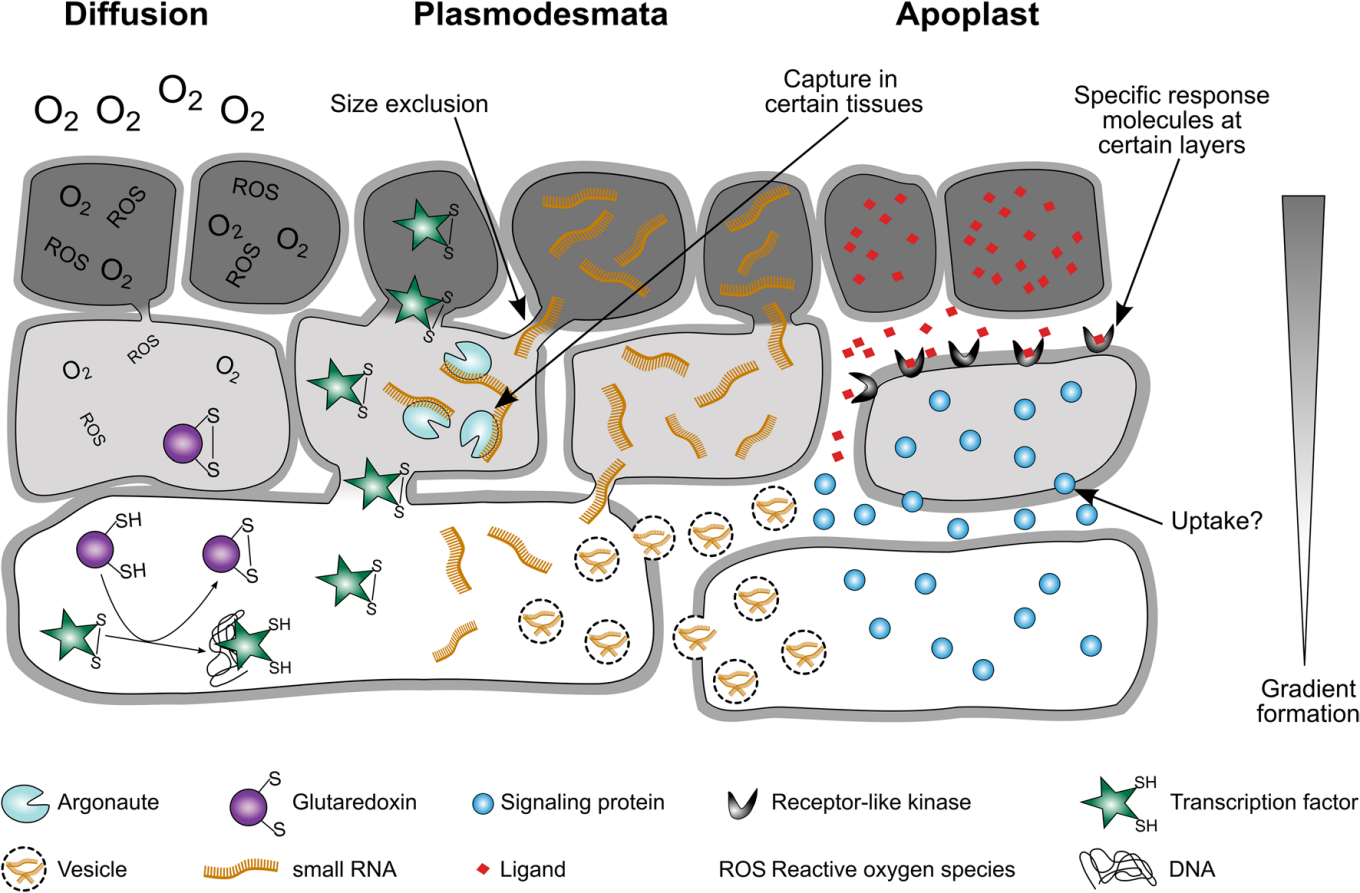
Schematic overview on proposed signal mobility and specificity in cell–cell communication. Some signals can freely diffuse (O_2_), while others migrate through plasmodesmata, e.g., transcription factors and small RNAs. Also, small RNAs might be transported in vesicles between cells. Proteins and ligands are secreted by unconventional and conventional secretion into the plant apoplast. There they can interact, for example, with receptor-like kinases or might be taken up by other cells. All these modes of mobility can result in signal gradient formation along tissues. Mobility can be limited by size exclusion of plasmodesmata or capture in certain tissues. Specificity of signals can be achieved by expression of response molecules in certain cell layers

**Fig. 3:**
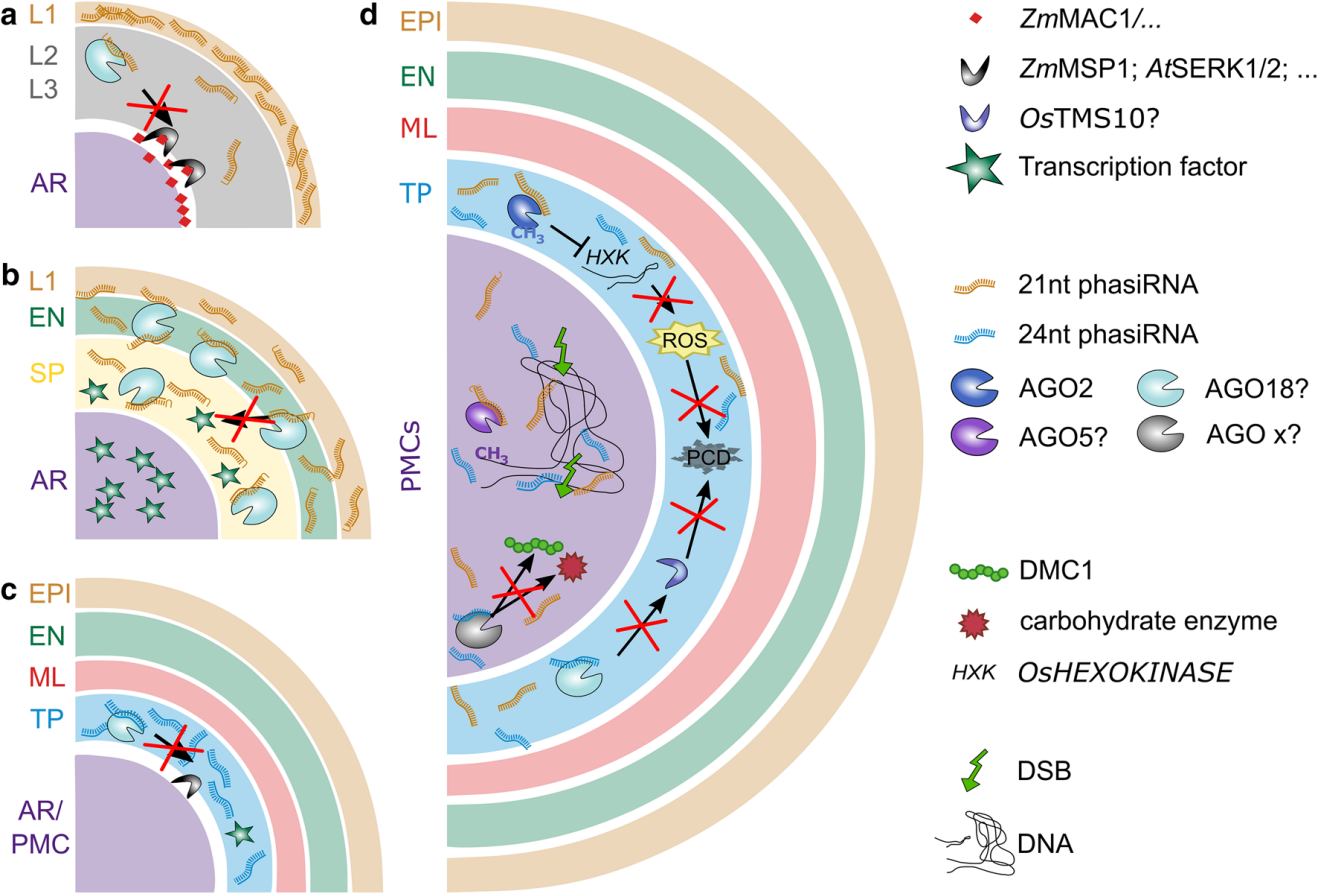
Speculative interconnection of mobile signals during anther development. **a, b** 21nt phasiRNAs are generated in the epidermis early in lobe differentiation. Consecutively, they will migrate toward the lobe center, load onto AGO18, and presumably degrade mRNAs for, e.g., **a** RLKs or **b** transcription factors in the emerging cell layers. **c, d** 24nt phasiRNAs emerge later, when all cell layers are formed, and derive from the tapetum. Both 21nt and 24nt phasiRNAs accumulate in PMCs and the surrounding tapetum. In the tapetum, they might be involved in timely initiation of PCD via gene silencing, by, e.g., DNA methylation of the HXK promoter, and via degradation of RNA for, e.g., RLKs involved in PCD initiation. Once phasiRNAs decrease, PCD could then occur. In PMCs, phasiRNAs seem to mediate DNA methylation, and thus/or independently promote early meiosis initiation with DSBs. DSB: double strand break, AR: archesporial, EN: endothecium, SP: secondary parietal, ML: middle layer, TP: tapetum, PMCs: pollen mother cells; ROS: reactive oxygen species, PCD: programmed cell death, HXK: *HEXOKINASE*

For very detailed insight into especially early anther development, we refer to previous reviews (Walbot and Egger 2016; van der Linde and Walbot 2019). What is currently poorly addressed is how the already known and partly characterized signals, namely transcription factors, ROS, peptides, and small RNAs, interact with and depend on each other. Here, we shortly lay out steps in formation of anther morphology (Fig. 1a), recapitulate knowledge about mobile elements (Table 1), and then offer some conclusions. The main focus of this article is to provide testable hypotheses (Figs. 2, 3) plus further questions with emphasis on ROS, secreted peptides, and small RNAs which could help to direct future research in the field.

## Morphology of anther development

In most angiosperm anthers, four lobes surround the connective tissue and the central vasculature. Within each lobe, layers of sub-epidermal somatic cell types (endothecium, middle layer, and tapetum) encircle the centrally located archesporial cells (AR cells), which later become pollen mother cells (PMCs; “meiocytes” during meiosis) and then pollen (Fig. 1a, b). The somatic cell layers are often only one cell thick, and all four layers are needed for successful male germline development, pollen maturation, and dispersal in most angiosperms.

Anther development can be separated into two distinct stages: (1) establishment of anther layers, followed by meiosis, and (2) pollen grain differentiation with accompanying re-differentiation and programmed cell death of certain anther layers (Goldberg et al. 1993). All anther cells trace back to the floral meristem and are L1-derived (L1-d) for the epidermis and plant-type-dependent L-2d and/or L-3d for all sub-epidermal cells (Goldberg et al. 1993; Kelliher and Walbot 2011). Based on stamen primordium location, initial adaxial-abaxial stamen patterning is established. This pattern is then rearranged in the anther resulting in a cross pattern of domains, where outgrowths between domains differentiate into lobes. At lobe inception, L2-d cells are pluripotent (Fig. 1a, b). A subset of L2-d cells differentiates into archesporial (AR) cells first. AR cells stimulate their neighboring L2-d cells to adopt a somatic fate through periclinal cell division. This generates two layers, the endothecium (EN) and secondary parietal cells (SPCs), establishing the somatic niche. The SPCs start to divide periclinally, forming two additional layers, the middle layer (ML) and tapetum (TP). This step is followed by cell proliferation, and differentiation of AR cells into pollen mother cells (PMCs) (for detailed review on premeiotic anther development see Walbot and Egger 2016). Meiosis (microsporogenesis) begins with the expansion of the individual PMCs which then undergo two rounds of cell division producing tetrads of haploid microspores (for review see Mercier et al. 2015). Callose deposition starts to isolate PMCs from each other later in meiosis, ending with microspores enclosed by a unique callose cell wall. Also, at the end of meiosis, the ML is mechanically crushed or degraded, and then, there is a short relapse phase when the callose wall is degraded by enzymes secreted from the tapetum to release the individual microspores. At this stage, the developing pollen is coated by exine components secreted by the tapetum, namely lipase and glycine-rich proteins (Lu et al. 2020) Afterward, the tapetum undergoes programmed cell death (PCD), with its cell remnants forming the pollen kitt on the maturing pollen. A first mitotic division of the microspore without cytokinesis results in a vegetative and a generative nucleus. A second mitotic division of the generative cell gives rise to two sperm cells. Dependent on the plant species, pollen mitosis II occurs within the anther or later during pollen tube growth, resulting in tricellular or bicellular pollen, respectively. For pollen release, first the septum between the two lobes within a theca and then the epidermis are degraded (for review see Hafidh et al. 2016). Obviously, all these developmental steps need a high degree of spatiotemporal coordination to allow proper development of the anther and the pollen within it.

## Challenges of anther development

Stamens lack a general central organization unit, i.e., meristem, and cells must self-organize. Curiously, stamens are considered modified leaves, with the same need to generate adaxial-abaxial patterns though in a more complicated fashion (reviewed in Walbot and Egger 2016). The lack of a meristem is reminiscent of the situation in both plant and animal embryos where internal polarization and positional cues serve to create basal/apical or anterior/posterior ends, respectively, with support by asymmetric cell divisions in self-organization (reviewed in Peris et al. 2010; White et al. 2018). In contrast, plant meristems contain stem cells that initiate and organize their surrounding cell populations as in animal stem cells (reviewed in Heidstra and Sabatini 2014). However, similar to meristem-free organ development, key molecules include secreted peptides, their receptors, and transcription factors.

Since anther development requires proper timing of events, in correct order and often synchronized between layers, signals need to coordinate anther development in a spatiotemporal fashion: (1) giving each step in development sufficient time while moving on to the next step as soon as possible, (2) ensuring the formation and development of separate layers, and (3) coordinating simultaneous progress in, e.g., tapetal development and PMCs (pollen mother cells).

## Mobile elements in anther development

Multiple mobile elements or their gradients along the anther radius shape anther development: Here, we focus on oxygen, secreted peptides and small RNAs (Fig. 1c).

Plant hormones are suitable candidates for shaping anther development as well, and seem to act especially very early and later than the core morphology-forming stages. A recent review covers the roles of gibberellin and jasmonate in stamen development (Marciniak and Przedniczek 2019). For example, gibberellic acid and jasmonic acid play roles in pollen formation and release via anther dehiscence (Zhao and Ma 2000; Cheng et al. 2004). Similarly, ethylene production peaks at degeneration of tapetum and middle layer, and at pollen maturation and dispersal (Kovaleva et al. 2011). Auxin participates in anther development, dehiscence, pollen maturation and filament elongation via ARFs (AUXIN RESPONSE FACTORS) (Nagpal et al. 2005; Wu et al. 2006; reviewed in Cardarelli and Cecchetti 2014) which will be further mentioned as small RNA targets below. Furthermore, Brassinosteroids have also been described in connection with ROS regulation in anther development as detailed below (Yan et al. 2020). In contrast, during early anther development, signaling is not mainly based on phytohormones (Walbot and Skibbe 2010; Zhang et al. 2014; van der Linde and Walbot 2019) but rather on environmental clues and other small mobile signals which orchestrate cell–cell communication (Kelliher and Walbot 2012; Zhang and Yang 2014; van der Linde et al. 2018b).

## Oxygen

In developing monocot anthers, hypoxic conditions are a natural result of rapid cell proliferation in flower organs and of the flowers being tightly encased by a whorl of not-yet-photosynthetic leaves. Consequently, an oxygen gradient is formed within the anther (Fig. 1c). In maize, this oxygen gradient can be manipulated by flushing the tassel buried in the leave whorl with O_2_ or N_2_ (Kelliher and Walbot 2012). Treatment with nitrogen shifted the hypoxia gradient more toward the outer cells of the lobes, where then ectopic AR cells specified. AR formation also dislocated by manipulation of redox status within the anther through treatment with reducing or oxidizing agents. Thus, oxidizing agents bias AR formation to the connective and vascular tissues, whereas reducing agent treatment results in subepidermal or epidermal ectopic AR cells (Kelliher and Walbot 2012).

The maize mutant with the earliest developmental defect after anther primordial formation is *Zmmsca1* (*male sterile converted anther1*) (Chaubal et al. 2003; Albertsen et al. 2011; Kelliher and Walbot 2012). In this mutant, L2-d cells fail to progress to archesporial fate and instead undergo longitudinal divisions and differentiate as vasculature but can be rescued by treatment with reductive agents (Kelliher and Walbot 2012). *ZmMSCA1* encodes a CC-type glutaredoxin, and homologs of *ZmMSCA1* have been identified in rice (*MICROSPORELESS1*, *OsMIL1*) and the dicot plant *Arabidopsis thaliana* (*AtROXY1* and *AtROXY2*) (Xing and Zachgo 2008; Hong et al. 2012a, b). *Osmil1* mutant development is disrupted after archesporial, endothecium, and secondary parietal layer formation (Hong et al. 2012b). Deletion of *Atroxy1* and *Atroxy2* prevents formation of normal parietal cells as well as pollen release, while meiosis is still induced (Xing and Zachgo 2008). It thus seems that the mutant defects occur at different developmental times and differentiation steps, maybe due to differences in the spatiotemporal redox gradients among species. Several studies provide evidence that CC-type glutaredoxins modify bZIP transcription factors of the TGACG (TGA) motif-binding family, thereby regulating their activity and downstream processes (Xing et al. 2005; Ndamukong et al. 2007; Li et al. 2009, 2011; Murmu et al. 2010; Hong et al. 2012b).

While oxygen and subsequently ROS levels are extremely low during early anther development, and by that trigger AR formation, they increase strongly when the middle layer and tapetum are formed (Yang et al. 2018), and are elevated when tapetum cell death occurs (Fig. 1c). Even though ROS have functions in signaling, at this stage their destructive potential toward proteins, DNA, and lipids seems to ultimately cause tapetal cell death (for detailed review about ROS in rice anther development see Yu and Zhang 2019). Tapetal cell death and thus ROS production need to be precisely timed to allow proper pollen formation. This seems to be achieved by at least two kinds of mobile elements, small RNAs and a phytohormone: ROS-mediated tapetal PCD is brassinosteroids (BR) and most likely small RNA dependent by modulation of ROS producing enzyme expression. Tomato BRASSINAZOLE RESISTANT 1 (*Sl*BZR1), a BR signaling regulator, directly binds to the promoter of *SlRBOH1 (RESPIRATORY BURST OXIDASE HOMOLOG)* and by that increases *Sl*RBOH1-dependent ROS production (Yan et al. 2020). Besides *Sl*BZR1, the argonaute *Os*AGO2 was shown to modulate expression of an enzyme involved in tapetal ROS generation. *Os*AGO2 facilitates DNA methylation in the promoter region of *Os*HXK (hexokinase) and by that subsequent ROS homeostasis is controlled. The Os*ago2* mutant is defective in anther development, and downregulation of *OsAGO2* causes ROS overaccumulation and premature PCD of the TP (Zheng et al. 2019b).

Taken together, anther development seems to make good use of the naturally occurring spatiotemporal oxygen/ redox gradient across anther lobes. In the beginning, when the anther primordia and surrounding leaf whorl are very dense, only low oxygen levels exist, which enable signaling. Later, oxygen and ROS levels increase in the anther, and ROS (likely specifically produced at certain layers like the tapetum) can then trigger PCD to release pollen from the anther.

## Small, secreted proteins

Besides the above-mentioned signals that induce ROS accumulation, tapetal degeneration seems to be also regulated via signaling by ligand–receptor interactions, as there are two rice RLKs (receptor-like kinases), *Os*TMS10 and *Os*TMS10L (THERMO-SENSITIVE GENIC MALE STERILE 10 and ~ -LIKE) that are needed for tapetal degeneration and male fertility, especially at high temperature (Fig. 1c) (Yu et al. 2017). Several more RLK mutants have defects in anther development, resulting in male sterility (for reviews see Zhao 2009; Kelliher et al. 2014; Cai and Zhang 2018). These are *Ater/erl1/erl2* (*erecta/* ~ *like1/2*) triple mutants with absent or defective anthers (Hord et al. 2008), *Atserk1/serk2* (*somatic embryogenesis receptor-like kinase1/2*) double mutants with excess AR cells and absent tapetum (Colcombet et al. 2005), *Atrpk2* (*receptor-like kinase2*) which lacks the ML (Mizuno et al. 2007), and *Atbam1/bam2* (*barely any meristem 1/2*) double mutants where mutant anthers have larger but fewer L2-d cells, only form AR-like cells and no internal somatic cells, and have shriveled appearance (Hord et al. 2006). *At*SERK1 and 2 were proposed to form heterodimeric receptors with *At*EMS1/EXS since their mutant phenotypes are the same (Colcombet et al. 2005). Proteomic data revealed more RLKs in developing anther cells (Ye et al. 2015, 2016), and their ligands are assumed to be small secreted proteins, but not yet identified. Potential ligand candidates can be found in the CLE (CLAVATA3/ESR-RELATED) group, for example. This idea is based on GUS-expression reporter lines in *A. thaliana*, which found six *AtCLE*s to be expressed in anthers: *AtCLE1* in the tapetum and in pollen grains, *AtCLE7* in anther lobes, *AtCLE11* in mature pollen, *AtCLE12* in all stages of pollen development, *AtCLE13* in young anthers, and *AtCLE25* throughout anther development in lobes (Jun et al. 2010).

The only ligand–receptor pair identified in anther development so far has been reported in both monocots and dicots: *At*TPD1 (TAPETUM DETERMINANT (1): *At*EMS1/EXS (EXCESS MALE SPOROCYTES 1/ EXTRA SPOROGENOUS CELLS) in *A. thaliana*, *Os*TDL1A (TPD1-LIKE 1A): *Os*MSP1 (MULTIPLE SPOROCYTE1) in rice, *Zm*MAC1 (MULTIPLE ARCHESPORIAL CELLS 1): *Zm*MSP1 in maize (Fig. 1c). Although cytological phenotypes differ among plant species, all receptor knockout mutants are male sterile (Sheridan et al. 1999; Nonomura et al. 2003; Yang et al. 2003, 2016; Wang et al. 2012a; Huang et al. 2016; van der Linde et al. 2018a). In maize, newly specified AR cells secret *Zm*MAC1 in its 218 aa form, which is recognized via the RLK *Zm*MSP1 of the surrounding L2-d cells (Wang et al. 2012a; van der Linde et al. 2018a). L2-d cells undergo a periclinal division and acquire the cell fate to form EN and SPCs when receiving the *Zm*MAC1 signal (van der Linde et al. 2018a). Notably, each individual L2-d cell has to receive the *Zm*MAC1 signal to facilitate this step as it was shown by microinjecting *Zm*MAC1 into the anther cell apoplast using the Trojan horse method (van der Linde et al. 2018a,b; Fiedler et al. 2019). This might mean that target cells are either not connected with each other, or that downstream signals and components are not transmitted via plasmodesmata (Fig. 2).

## Small RNAs

Peptides are well known as morphogens, acting by forming a gradient. Small RNAs (sRNAs) might play similar roles, as suggested by Benkovics and Timmermans (2014). Not many specific sRNAs regulating anther development are known but is clear from the characterized miRNAs (see below) that they play important roles. Similar to miRNAs, tasiRNAs (trans-acting small interfering RNAs) have specific target genes which they regulate by mRNA degradation. tasiRNAs are a subclass of phasiRNAs, which are named according to their biogenesis since they are processed from longer dsRNA precursors into consecutive fragments of 21 or 24nt length. Another phasiRNA subclass, i.e., highly abundant reproductive phasiRNAs in mainly monocots, has the potential to include further yet undescribed tasiRNAs supporting anther development (Zhai et al. 2014).

### miRNAs

A few key miRNAs for anther development have been studied, but for most of them it is unknown whether they are mobile between cells. The general importance of miRNAs for anther development is evident through mutants of the miRNA biogenesis factor DCL1 (DICER-like1). The maize *Zmdcl1* mutant “*fuzzy tassel*” is defective in miRNA biogenesis and is male sterile due to defects in late anther maturation and dehiscence (Field and Thompson 2016). The Arabidopsis *Atdcl1* mutant has still viable pollen but partly absent or abnormal anthers with less than four lobes (Jacobsen et al. 1999). Specific miRNAs generated by *At*DCL1 might thus play roles in early anther formation and later anther maturation and dehiscence, for example, miR165/6, miR167 and miR156 as described below.

miR165/6 is a specific miRNA with roles in anther development, and its expression patterns suggest that it is mobile. Before lobe initiation, miR165/6 accumulates in lateral-adaxial regions of stamen and then in the four newly formed corners and afterward—but before TP differentiation—in the four lobe centers (Li et al. 2019a). MiR165/6 overexpression in Arabidopsis highlights the early role of this miRNA in shaping the anther structure through the formation of internal boundaries since only two instead of four lobes emerge (Li et al. 2019a). Additionally, studies in Arabidopsis showed that MiR165/6 targets *At*PHB (PHA-BU-LO-SA) which in turn binds to the promoter of *At*SPL/NZZ (SPOROCYTELESS/NOZZLE) to activate it for its role in microsporogenesis (Li et al. 2019a). In *Atspl/nzz* mutants, EN and TP cannot form, and AR cells do not progress to meiosis (Yang et al. 1999; Liu et al. 2009b).

Mobility between cells has been suggested for *At*miR167, which seems to be expressed in vascular cells, but retained in connective cells (Wu et al. 2006). If its targets, *At*ARF6 and 8 (AUXIN RESPONSIVE FACTOR) are immune to downregulation by miR167, connective cells become very large, locules do not break, and no dehiscence occurs (Wu et al. 2006). The same is the case for *At*miR167 null mutants, and together with other data, this led to the model where miR167 arrests growth to permit for more time for anther dehiscence (Zheng et al. 2019a).

Another miRNA in early anther development, *At*miR156, targets transcription factors of the SPL (SQUAMOSA PROMOTER BINDING PROTEIN-LIKE) family which in turn regulate genes for cell division, differentiation and specification (Xing et al. 2010). Expression of *At*miR156 arises dynamically from multiple loci, but mobility and source and sink cells have not yet been addressed.

### tasiRNAs

Anthers need to initially establish adaxial-abaxial patterns, which are known to be shaped by gradients of specific tasiARFs during leaf development (Chitwood et al. 2009). Similarly, a rice mutant of the tasiRNA production enzyme *Os*SHL2 (SHOOTLESS 2, an RNA-dependent RNA polymerase) has adaxial-abaxial anther patterning defects (Toriba et al. 2010), pointing to the use of tasiRNA gradients as a general developmental strategy in different organs, including anthers.

Until very recently, no specific tasiRNAs have been implied in reproductive processes, but vegetative phasi/tasiRNA targets indicated that reproductive phasi/tasiRNAs might be good candidates to regulate processes in anther development as well: (1) TasiRNAs from disease-related NB-LRR loci regulate NB-LRRs and thus subsequent PCD (Zhai et al. 2011; reviewed in Cui et al. 2015), (2) tasiRNAs target transcription factors of the NAC and MYB classes (reviewed in Liu et al. 2020), and, most importantly, (3) a recent study in tomato revealed tasiRNAs/non-reproductive phasiRNAs to target multiple RLKs (Luan et al. 2020). In reproductive tissues, most studies could either not predict or prove that phasiRNAs target and degrade other mRNAs (Song et al. 2012; Zhai et al. 2015; Patel et al. 2018). One study in pummelo predicted and verified targets *in trans* with gene ontologies of broad terms but also including PCD and pollen development (Fang et al. 2020). By now, two pioneering study in rice meiocytes finally found indeed RNA degradation of target genes of reproductive 21nt-phasiRNAs (Jiang et al. 2020; Zhang et al. 2020c). In rice meiocytes, target genes were enriched for carbohydrate biosynthesis and metabolism, and also included *OsDMC1B*, a meiotic gene (Jiang et al. 2020). Another study gained insight on genes targeted for degradation in rice anthers through degradome data from whole rice spikelets of WT and of mutants in MEL1, the rice AGO5 which binds phasiRNA. Intriguingly, many RLKs were identified as phasiRNA target genes, among them MSP1 (Zhang et al. 2020c). This re-defines part of the reproductive phasiRNAs as tasiRNAs, with specific target genes with roles and/or high abundance in meiosis and anther development (Dukowic-Schulze et al. 2014; Wang et al. 2016).

### Reproductive phasiRNAs

Reproductive phasiRNAs are expressed at very high abundance in mainly monocot anthers, and arise from several hundred genomic locations (PHAS loci). Their biogenesis and occurrence throughout anther development and phyla have been addressed in several studies (Johnson et al. 2009; Zhai et al. 2015), and the current knowledge on phasiRNA has been comprehensively reviewed in Liu et al. (2020). In short, there are two waves of phasiRNA expression, distinguishable by their size (21 vs. 24nt), peak in abundance (premeiotic vs meiotic), and biogenesis factors (miR2118 vs. miR2275; DCL4 vs DCL5) as seen in rice and maize (Johnson et al. 2009; Song et al. 2012; Zhai et al. 2015). Interestingly, phasiRNAs from an individual PHAS locus do not have the same abundance, which is likely due to stabilization of only functionally relevant phasiRNAs by, e.g., AGO proteins. Indeed, *Os*MEL1 (MEIOSIS ARRESTED AT LEPTOTENE 1; rice AGO5) binds 21nt phasiRNAs with mainly 5′C in PMCs (Komiya et al. 2014), and *ZmAGO18b* binds 21nt phasiRNAs with mainly 5′U (Sun et al. 2019) which occur preferentially in anther wall cells as shown in rice (Araki et al. 2020). Whether AGO proteins bind to these phasiRNAs at the place of action or whether they migrate as bound complexes is unresolved. The fact that 21nt phasiRNAs seem to bind to different AGOs implies that different molecular mechanisms such as PTGS (posttranscriptional gene silencing) or RdDM (RNA-dependent DNA methylation) could be mediated by phasiRNAs.

Arikit et al. (2013) suggested that 21nt phasiRNAs might be involved in PTGS (see “tasiRNA” section above) and 24nt phasiRNAs in silencing by RdDM. DNA methylation has indeed been shown *in cis* at maize PHAS loci, albeit at both 21 and 24nt PHAS loci, and recently been confirmed with extensive analysis of maize phasiRNA mutants (Zhang et al. 2020b). Since increased DNA methylation was detected in meiocytes, a role in meiotic chromosome dynamics has been suggested (Dukowic-Schulze et al. 2016), similar to fission yeast lncRNAs that are involved in recognition and pairing of homologous chromosomes (recently reviewed by Hiraoka 2020). In line with a function of phasiRNAs in chromosome dynamics, a role for the phasiRNA-binding rice ARGONAUTE *Os*MEL1 in meiotic chromosome structure has been suggested, in addition to a role in male sporophyte development (Komiya et al. 2014). Rice *Osmel1* mutants have defects in development of tapetum and PMCs (pollen mother cells), arresting early due to problems during premeiotic mitosis or male meiosis where chromosome condensation and synapsis is defective (Komiya et al. 2014). Furthermore, *Os*MEL1 localization was detected in the nucleus in meiosis and in the cytoplasm otherwise (Komiya et al. 2014).

## Means to achieve mobility and specificity

As apparent from Fig. 2, three major ways of signal mobility have been reported in anther development: (1) diffusion, (2) migration through plasmodesmata and cytomictic channels, and (3) via the apoplast. PMCs during microsporogenesis are connected by cytomictic channels through which even nuclear material, i.e., chromosomes, can migrate (reviewed in Mursalimov et al. 2013). These special cellular connections have been observed for over a century, and also have been reported between TP cells during pollen maturation; the smaller and more widespread plasmodesmata, on the other hand, connect multiple somatic anther cells with each other. Plasmodesmata between PMCs and TP cells exist and become closed when pollen mature, so that TP cells are isolated when they undergo PCD (reviewed in Sager and Lee 2014). Plasmodesmata also connect EN cells with each other, and with EPI and ML cells (Mamun et al. 2005).

A beneficial property for elements to be mobile is their relatively small size. Small molecules like O_2_ move across cell membranes via diffusion resulting in gradient formation, which is dependent on factors like temperature, and consumption in certain tissues as it was proposed in anther development (Kelliher and Walbot 2012). In contrast to this, ROS have a strongly limited mobility because of their short lifespan and by that can function mostly at their origin as signals.

A limiting factor for symplastic migration is plasmodesmata size which is, for example, modulated via redox regulation (Benitez-Alfonso et al. 2009; reviewed in Maule et al. 2011), and can change depending on the developmental state. Immature cells seem to have increased transport rates, for example, in Arabidopsis embryos up to the torpedo stage when 0.5 kDa tracers can still pass but 10 kDa tracers are excluded (Kim et al. 2002; reviewed in Brunkard et al. 2013). Intriguingly, the above-mentioned RLKs *At*BAM1/BAM2 have been implied in increasing plasmodesmata size in the context of RNAi spread as part of antiviral defense (Rosas-Diaz et al. 2018).

Proteins and peptides can be secreted into the plant apoplast via conventional or unconventional secretion. So far, only one apoplastic ligand was identified in anther development, namely *At*TPD/*Os*TDL1/*Zm*MAC1, which contains a signal peptide sequence for conventional secretion (Wang et al. 2012a). *Zm*MAC1 was shown to be secreted by onion cells, and later experiments confirmed that ZmMAC1 acts in the maize anther apoplast (Wang et al. 2012a; van der Linde et al. 2018a).

The ability of tasiRNA/phasiRNAs to migrate across cell layers seems to be a core property for reproductive phasiRNAs. 21nt phasiRNAs are generated in outer layers like the epidermis and end up later at highest concentrations in tapetal cells and PMCs (Fig. 3a, b, d), similar to 24nt phasiRNAs that are generated later in the TP (Fig. 3c, d) (Huang et al. 2020). How phasiRNAs migrate from cell to cell within the anther remains elusive. In Arabidopsis leaves, phasi and tasiRNAs have been found in extracellular vesicles (Baldrich et al. 2019), while in the plant developmental context, sRNAs were described to travel through plasmodesmata (Dunoyer et al. 2013).

The spatiotemporal specificity of mobile signals is an essential factor in cell–cell communication and dependent on multiple aspects, for example, on pace: tasiRNAs seem to be faster in their cell-to-cell-movement than miRNAs (Felippes et al. 2011), maybe because tasiRNAs are generated in the cytoplasm but miRNAs mainly in the nucleus, with the nuclear export rate as a limiting factor (Jouannet et al. 2012; reviewed in Rogers and Chen 2013). Developmental sRNAs seem to migrate less far than somatic sRNAs (3–6 vs 10–15 cells) (reviewed in Benkovics and Timmermans 2014). While sRNA gradient formation might just be an unavoidable result of their mode of migration within an organ, their tissue-specific signaling function could dependent on either the “Threshold Model” or the “Gradual Model” (reviewed in Benkovics and Timmermans 2014). On the other hand, specificity at that point might be achieved by binding to a tissue-specific AGO protein with preference for 5′ uridine 21nt phasiRNAs in anther cell wall (Araki et al. 2020) or 5′ cytosine 21nt phasiRNAs in PMCs (Komiya et al. 2014). Binding to a partner such as an AGO protein is a prominent way to prevent further spreading of sRNAs, as reported for AtAGO10 binding to miR166 stopping it from moving to the embryonic meristem (Liu et al. 2009a; Zhu et al. 2011). These partners could also be lncRNAs, so-called ceRNA (competitive endogenous RNAs) whose purpose is to capture sRNA at certain times and position before or after their actual action (Li et al. 2019b). In turn, sRNAs could act in supporting mRNA decay of genes before or after their time of need, presumably key factors such as transcription factors, and RLKs and by this support spatiotemporal specificity of other mobile elements (Fig. 3c).

Thus, mobile elements should always be considered together with the presence of their potential partners, and this applies to tasi/phasiRNA-target pairs as well as peptide ligand–receptor pairs, and redox-sensitive transcription factors.

## Open questions (Q) and hypotheses (H)

Proper anther and pollen formation are essential for fertilization and by that a key factor in agriculture. Several mobile signals that govern anther development have been identified (e.g., see Table 1), but many questions about their functions remain open.

### Why are anther developmental modules conserved but not consistently so?

- Q1: Why do conserved signaling modules (such as *Os*MIL1/*Zm*MSCA1/*At*ROXY1/*At*ROXY2 and *At*TPD/*Os*TDL1/*Zm*MAC1, together with their interactors) have different effects in anther development according to their mutant phenotypes? Specifically, the mutant phenotypes of *Zmmsca1*, *Osmil1* and *Atroxy*1/2 occur early in AR specification, later after EN/SP differentiation, at TP/SPC differentiation and pollen release, respectively.
  - H1: Answers to differences due to *Os*MIL1/*Zm*MSCA1/*At*ROXY1/*At*ROXY2 might lie in small changes in the respective oxygen gradients and migration properties. For example, Arabidopsis, rice and maize anthers are of different size and in vastly different surroundings during the formation. Besides differences in environmental conditions, expression patterns of the glutaredoxins or their targets might differ or their mobility between tissues. For the secreted peptide homologs *At*TPD/*Os*TDL1A/*Zm*MAC1, their spatiotemporal presence influenced by mobility and turnover could differ.
- Q2: Why do reproductive phasiRNAs seem to be highly conserved in monocots but less so in angiosperms (Xia et al. 2019; Feng et al. 2019)?
  - H2: At the moment, it can only be speculated that phasiRNAs are either not essential, or that their functions are taken over by other small RNAs that follow a different biosynthesis path but have similar functions.

### How do reproductive phasiRNAs contribute to anther development?

- Q3: How can the class of phasiRNAs mediate different outcomes? Is DNA methylation in *cis* a side effect or does it have a real biological function (Fig. 3d)?
  - H3: The first part is already partly resolved, i.e., by binding to different AGO proteins at certain layers and time. The second part needs further examination.
- Q4: What are the functions of genes targeted by phasiRNAs for degradation, as identified now in rice (Jiang et al. 2020; Zhang et al. 2020c)? Are there further targets in other cells, at other times? Are phasiRNAs maybe involved in the processes mediated by AGO2 in tapetal PCD, or by DCL1 in early anther formation or late anther dehiscence? Why might the regulation of targets be needed, and most so when the developmental timing is altered?
  - H4: Undoubtedly, monocot-prevalent reproductive phasiRNAs do play a supportive, but maybe non-essential role in anther development and/or meiosis, since mutants lacking the otherwise vast abundance of 21 or 24 nt phasiRNAs have phenotypes including defects in meiosis and anther morphology, as well as environment-sensitive male sterility (Liu and Nonomura 2016; Sun et al. 2018; Ono et al. 2018; Teng et al. 2020). This can occur through temperature or photoperiod, depending on the kind and amount of phasiRNAs missing or misregulated, and species and genetic background (Ding et al. 2012a, b; Zhu and Deng 2012; Fan et al. 2016; Teng et al. 2020). Common to both photoperiod and temperature changes is the modification of developmental timing—higher temperature as well as long-day conditions lead to faster anther development, meiosis and microsporogenesis (Zhang et al. 2020a; Zhu et al. 2020; Teng et al. 2020). One hypothesis regarding the biological function of phasiRNAs is a role in destabilization of mRNA that are not needed anymore, as by animal piRNAs (Gou et al. 2014). Given enough time, this RNA decrease will occur naturally, but in conditions that accelerate development, phasiRNAs might be needed.
- Q5: Could phasiRNAs coordinate anther development and meiosis simultaneously, enabling proper and coordinated timing of, e.g., tapetum redifferentiation and PMC stage?
  - H5: Reproductive tasi/phasiRNAs are indeed good candidates for supporting anther development by any of the following possibilities which remain to be verified: (1) supporting decay of mRNAs not needed anymore, by (2) building transcription factor gradients, by (3) removing other anther layer specifying factors like receptors or oxygen sensors at a certain time and/or from certain layers, and/or by (4) changing the chromatin state of meiotic chromosomes to support pairing or other processes.
- Q6: Are phasiRNAs influencing DSBs (double strand breaks) which are initiated in meiosis on purpose? This question stems from the observations that the usual consequences of DSBs, γH2AX marks on chromosomes, and high expression of the DSB repair mediator ATM (ATAXIA TELANGIECTASIA MUTATED) are decreased if 21nt phasiRNA pathways are defective (Liu and Nonomura 2016; Teng et al. 2020).
  - H6: There is no answer yet, but two possible, already shown, pathways that could contribute: increase in DNA methylation on meiotic chromosomes (Dukowic-Schulze et al. 2016; Zhang et al. 2020b) and regulation of meiotic proteins via RNA degradation (Jiang et al. 2020).

### How do known mobile elements in AR cell formation act exactly?

- Q7: Why (or how) do L1-d cells have such a high degree of plasticity that they can become AR cells under certain redox-altering conditions?
  - H7: The spatiotemporal location of *Zm*MSCA1 might be an aspect and needs further detailed investigation.
- Q8: Why do pluripotent L2-d cells surrounding the AR cell column acquire a somatic fate, in spite of a mild hypoxia and *Zm*MSCA1 expression (both of which promote AR cell formation)?
  - H8: Here, two possible scenarios come to mind: (1) The required hypoxia threshold is reached first in future AR cells, rapid differentiation and subsequent secretion of ZmMAC1 hinders surrounding cells. (2) Even though L2-d cells are morphologically identical (Kelliher et al. 2014), they might differ in expression and by that in responsiveness toward hypoxia. Initial patterning of stamen, anthers and anther lobes is based on expression domains within subsets of L2-d cells (Toriba et al. 2010) and might continue during AR and somatic cell differentiation.
- Q9: How does *Zm*MAC1 limit the number of AR cells?
  - H9: There might be yet undiscovered, subsequent signaling from the newly specified EN/SP cells involved. On the other hand, *Zm*MAC1 might interact with a receptor on the AR surface, maybe even *Zm*MSP1, and by that control AR cell number.
- Q10: Why is there *ZmMAC1* expression detectable before and after (even during meiosis) AR cell initiation?
  - H10: While a function of *Zm*MAC1 before AR differentiation is not promoted by mutant analysis, one might speculate that *Zm*MAC1 functions as a reoccurring signal later in anther development. The strong, initial AR and somatic layer phenotype observed in mutants may cover-up later phenotypes. Inducible mutants or further studies using the Trojan horse approach on different stages of anther development might help to answer this question.
- Q11: How are mobile signals transmitted toward transcription factors and vice versa?
  - H11: One way might be by direct interaction of signal receptor with transcription factors. It has been suggested that the naturally occurring oxygen gradient is sensed by *Zm*MSCA1/*Os*MIL1 within the anther, accompanied by reduction of its disulfide bridge (see Fig. 2). In turn, *Zm*MSCA1/*Os*MIL1 could reduce and by that activate TGA-transcription factors (Fig. 2), resulting in lobe cell specification.

### Which yet unidentified factors and functions are we still missing?

- Q12: How many apoplastic peptide/protein signaling pathways are yet to be discovered in anther development?
  o H12: A variety of proteomic analysis identified RLKs and potential ligands in developing anther cells (Wang et al. 2012b; Zhang et al. 2014; Ye et al. 2015, 2016)) but, only a few have been studied yet, and ligands for most known receptors remain unknown. Another indication for the impact of apoplastic proteins/signals in anther development is the importance of a signal peptide peptidase (SPP) during pollen development in Arabidopsis. SPPs are endoplasmatic reticulum localized proteases and cleave signal peptides from proteins entering the secretory pathway. The *Atssp* mutant shows poor pollen germination, and nuclear morphology is abnormal at the tricellular pollen stage. *AtSSP* expression is highest in root, shoot apex, carpel, and stamen/pollen as well as in stage 6 and 7 seeds (Han et al. 2009).
- Q13: How many other cell–cell communications pathways are we still missing in anther development?
  - H13: Apoplastic vesicles containing RNA (Baldrich et al. 2019; for review see Cai et al. 2019), direct uptake of proteins from the apoplast (for review see Petre and Kamoun 2014) and transport of bigger proteins through plasmodesmata (reviewed in Stahl and Simon 2013) have been discovered and shown to play pivotal roles in other cell–cell communication scenarios and could possible play roles in anther development as well.

### How are the mobile elements interconnected?

Figure 3 sums up suggested interactions between transcription factors and mobile signals (phasiRNAs, ROS, RLKs and secreted peptides), which, however, remain to be demonstrated. Two specific thoughts are the following ones:

- Q14: Are mobile signals connected with each other?
  - H14: A first indication for the connection of different mobile signals might come from mutant analysis. The maize *Zmocl4* (*outer cell layers 4*) mutant lacks 21nt phasiRNAs and has abnormal and partly duplicated endothecium layers, attributed to defective epidermal signaling (Wang et al. 2012a). If the 21nt phasiRNAs include tasiRNAs that target *Zm*MSP1 or *Zm*MAC1 for RNA degradation, *Zmocl4* mutants might have prolonged presence of *Zm*MSP1 or *Zm*MAC1, resulting in more cells to become EN (Fig. 3a).
- Q15: Which anther cell types produce the mobile signals (brassinosteroids and smallRNAs) that influence transcription of ROS production enzymes and when, and by that ultimately regulate ROS levels in the TP?
  - H15: Here, we speculate that AGO2 might use either 21nt or 24nt phasiRNAs, or both to mediate DNA methylation at promoters and thus prevent premature ROS bursts (Fig. 3d). This could explain the defects in tapetal PCD in *Zmdcl5* mutants which lack 24nt phasiRNAs (Teng et al. 2020).

## Summary and outlook

**Fig. 4:**
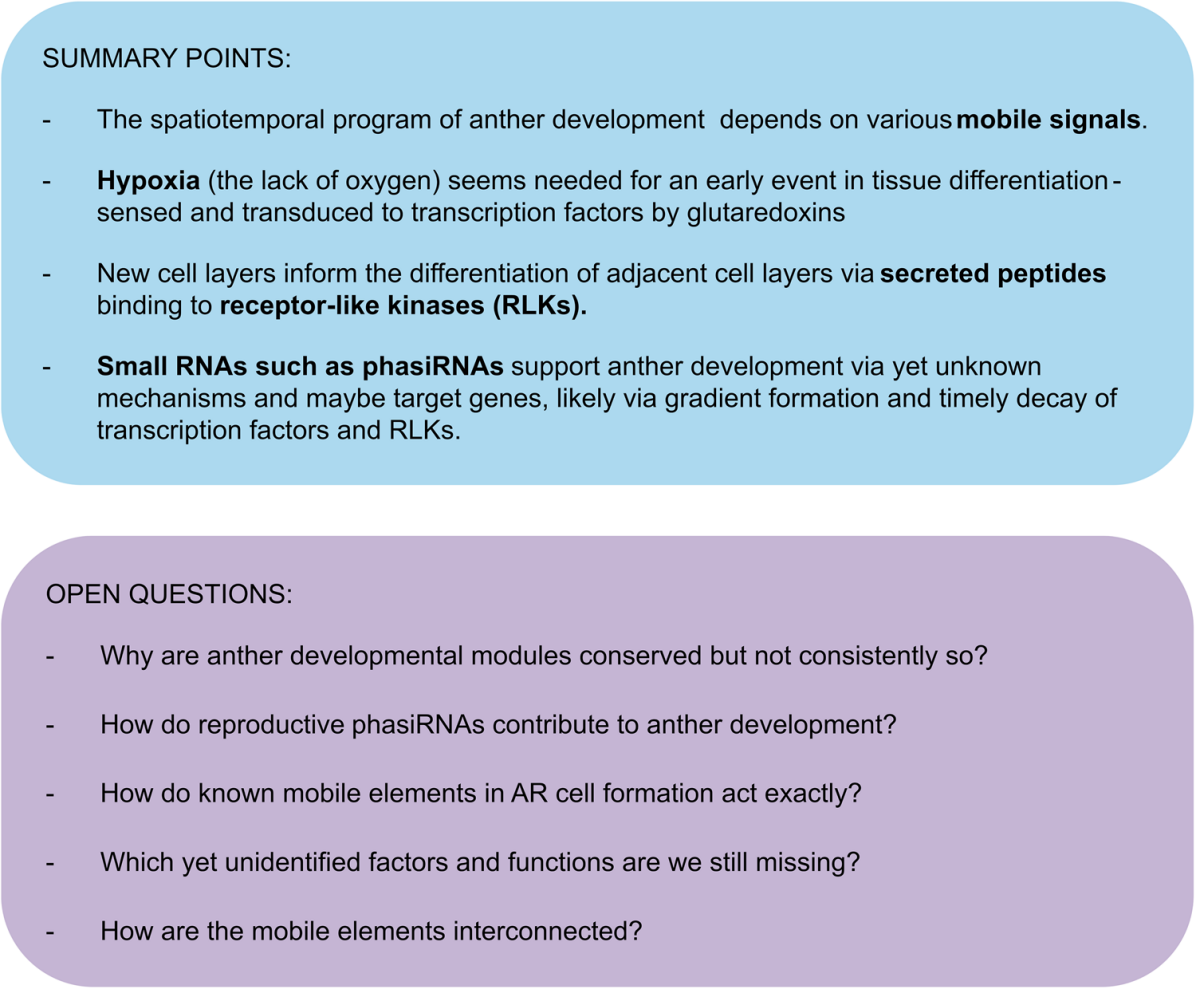
Summary points and open questions

Taken together, the triad of oxygen, secreted proteins and sRNAs are key players beside phytohormones governing the spatiotemporal program of anther development (Fig. 4). They arise naturally, or emerge from newly formed cell layers and can migrate from their location of origin along the lobe radius. While O_2_ naturally forms a gradient across the anther, the allocation of other mobile signals remains more elusive. Specific sRNA might form gradients, while other sRNAs might be retained in certain anther tissues. Mobile sRNAs could support timely and position-specific presence of components like transcription factors and RLKs and thereby interconnect with other mobile element pathways. Whether this turns out to be the case will need to be experimentally validated by using, e.g., sequencing approaches including AGO-immunoprecipitations to identify target genes, and phasiRNA-defective lines for cytological approaches such as (F) ISH to study regulation of these target genes. Proteomic data of anthers, among other published data, indicate that there are more protein signals to be found in the future. Spatiotemporal, functional and conservational characterization of those will help to understand their mobility, how they gain spatiotemporal specificity, and how mobile element pathways mingle to orchestrate anther development together. We look forward to future discoveries in the field of mobile elements whether they support our partly speculative hypotheses here or dispute them to move on to novel ideas.

### Authors contribution statement

SDS and KL equally contribute to this manuscript.

## Abbreviations

AR: Archesporial
PPCs/SPCs: Primary/secondary parietal cells
EPI: Epidermis
EN: Endothecium
ML: Middle layer
TP: Tapetum
PMC: Pollen mother cell
RLK: Receptor like kinase
ROS: Reactive oxygen species
